# Botanical Description of British Plants

**Published:** 1806-09

**Authors:** 


### *Botanical Description of British Plants.*

( Continued from pp. 165—171.)

Icosandria. Polygynia.

14. Rosa. *R. Spinosissima.*

*Ang.* Burnet rose. Pimpernel rose. *Var. with striped fl.* Ciphian rose.

*Gen. Desc.* Petals 5. Cal. urn-shaped, five-cleft, fleshy, contracted at the neck so as to form at length a coloured berry of one-cell, opening at top. Seeds many hispid, dispersed in the pulp.

*Spec. Desc.* *Germen* globular, smooth. *Stem* and leaf stalks thick set with awl-shaped, straight, horizontal, unequal prickles. *Leafits* small, circular, smooth, sitting. *Leafscales* small, halberd-shaped, toothed. *Cal. leaves* v. entire. *Bloss* white, cream colour, or red. Prickles sometimes flat. *Heaths and sandy places.* Bl. June, July.

*Use.* The ripe fruit is eaten by children: it has a grateful subacid taste. The juice of it, diluted with water, dyes silk and muslin of a peach colour: and with addition of alum, a deep violet; but it has very little effect on woollen or linen. Its dwarfish growth, and the singular elegance of its little leaves, which resemble those of the upland burnet, entitle it to a place in the flower garden.—*Withering.*

15. Rosa. *R. canina.—R. sylvestris vulgaris. Cynosbatus.*

*Ang.* Dog's rose. Hep tree. Wild briar.

*Gen. Desc.* *As above.*

*Spec. Desc.* *Germen* egg-shaped, smooth. *Stem* and *leaf-stalks* prickly. *Prickles* hooked downwards, broad, flat. *Leafits* 2 or 3 pair, with an odd one, pointed, serratures terminated by minute purple glands. *Leaf-stalks* sheathing; edges beset with purple glands. *Floral-leaves* 2, opposite fringed. *Petals* two-lobes, one larger, flesh-

 colour colour, or red, sometimes nearly white. *Hedges, woods.* Bl. June.

*Use.* The flowers of the damask rose are *purgative,* those of the red rose *astringent,* and the fruit of the wild rose *pectoral:* they are all used in the preparations of the shops.—*Hill.* The fruit, called hipps, or hepps, has a sourish taste, and obtains a place in the London Pharmacopœia, in the form of a conserve; for this purpose the seeds and chaffy fibres must be carefully removed, for if these prickly fibres be not entirely scraped off from the internal surface of the hepps, the conserve is liable to produce irritation on the primæ viæ. This preparation, though formerly esteemed useful in many disorders, as dropsy, calculous complaints, dysentery, hæmorrhages, &c. is not now supposed to possess any medical virtue, but being agreeable to the taste, is well suited to give form to more active articles of the Mat. Med.—The moss-like prickly excrescence, called Bedeguar, or rose sponge, by the French Galle chevelue, found upon the branches of this tree, was formerly in great repute as a medicine in various diseases: it is the habitation of an insect, *Cynips rosæ.*—*Woodville.* The pulp of the berries beaten up with sugar, makes the *conserve of hepps* of dispensatory: mixed with wine, it is an acceptable treat in the North of Europe. The leaves of every species of rose, but especially of this, are recommended in *Eph. Nat. Curios.* as a substitute for tea, giving out a fine colour, a sub-astringent taste, and a grateful smell, when dried, and infused in boiling water. A perfumed water may be distilled from the blossoms, which, though commonly described as inodorous, have a faint fragrance. An infusion of the full blown blossoms of all the roses, but especially of the paler kinds, is *purgative;* but the petals of the red roses, gathered before they expand, and dried, are *astringent.*—Several birds feed upon the berries.—*Withering.*

16. Rubus. *R. idæus.*

*Ang.* Raspberry bush, or bramble. Framboise. Hind-berry. Raspis.

*Gen. Desc.* Cal. five-cleft. Petals five. Styles from the top of the germens. Drupa clustered, one-celled, fixed to a conical receptacle so as to resemble a berry.

*Spec. Desc.* *Stem* shrub-like, upright, green, 2 feet high, biennial, bearing 2d year, prickly. *Leaves* winged, five or three leafits, serrated, cottony underneath. *Leaf-stalk* chanelled. *Fruit-stalk* hairy rough. *Bloss.* white. *Berry* red, or white. *Woods, hedges, moist mountains.* Bl. May, June.

*Use.* This fruit, which is commonly cultivated in our gardens, has a pleasant sweet taste, accompanied with a peculiarly grateful flavour, on account of which it is chiefly valued. Its medicinal virtues consist in allaying heat and thirst, and in promoting the natural excretions; but it seems less adapted to answer these purposes than many of the other summer fruits. A grateful syrup, prepared from the juice, is directed for use by the London Pharmacopœia.—*Woodville.* In the Isle of Skye the juice or a syrup of the fruit is frequently used as an agreeable acid for punch, instead of oranges or lemons. A distilled water from the fruit is cooling; and in *fevers* beneficial.—*Lightfoot.* The fruit, extremely grateful as nature presents it, is much improved in flavour by being made into a sweetmeat with sugar, or fermented with wine. It is fragrant, sub-acid, and cooling.—It dissolves the tartarous concretions of the *teeth,* but for this purpose, it is inferior to the straw-berry. The white berries are sweeter than the red, but they are generally contaminated with insects. The leaves are the favourite food of kids.—*Withering.*

17. Rubus. *R. fructicosus.* *R. vulgaris fructu nigro.*

*Ang.* Bramble. Blackberry-bush. Bumblekites.

*Gen. Desc.* *As above.*

*Spec. Desc.* *Stem* prickly, angular, very long, with runners spreading and climbing prodigiously, sometimes striking root. *Leaves* winged; 3 or 4 leafits, serrated, the uppermost on a leafstalk, the rest nearly sitting. *Prickles* alternate, strong, bowed back. *Petals* flaccid, white or purplish. *Fruit* black. *Hedges, woods.* Bl. June, Sept.

*Use.* A jam made of the berries has been found effectual in the *gravel* by the present Archbishop of Armagh, Ed. The berries when ripe are black, they have a pleasant flavor, and are commonly eaten by children: they taste well with wine.—The green twigs are of great use in dying woollen, silk and mohair, black.—*Withering.* Cows and horses eat it; sheep are not fond of it.—*Linn.* Silkworms will feed upon its leaves in defect of those of the mulberry. —*Stokes.*

18. Rubus. *R. chamæmorus.*

*Ang.* Cloud-berry. Bramble. Knot-berry. Knout-berry.

*Gen. Desc.* *As above.*

*Spec. Desc.* *Stem* scarcely 1 foot high, not prickled, with 1 flower: Diœcious. *Leaves* simple, lobed. *Bloss.* white white or purple. *Berry* red. *Peat bogs, sides of mountains.* Bl. May, June.

*Use.* The berries are not unpleasant, and are esteemed an excellent *anti-scorbutic,* especially in Sweden and Norway: from the latter, large quantities are sent, packed in wooden vessels, to Stockholm, where they are served up as deserts, or made into tarts. The Laplanders bury them under the snow, and thus preserve them fresh from one year to another, digging them out in the spring as good as when gathered; they bruise and eat them with the milk of the rein-deer. In the Highlands of Scotland, they are also brought sometimes to table with the desert.—*Withering.—Lightfoot.*

19. Fragaria. *F. vesca.*

*Ang.* Strawberry.

*Gen. Desc.* Cal. ten-cleft. Petals 5. Seeds naked, smooth, on a receptacle, which is egg-shaped, coloured, deciduous, resembling a berry.

*Spec. Desc.* *Leaves* by threes, egg-shaped, serrated. *Runners creeping,* long, slender, smooth, often purplish. *Leafstalks* woolly. *Bloss.* white. *Fruit* red, or white. *Woods, hedge banks.* Bl. May, June.

*Use.* The root and leaves are *astringent,* and have been used as a *vulnerary.* The fruits will dissolve the tartarous incrustations of the teeth without acrimony: they have also a *diuretic* quality, and are useful in the *stone* and *gravel.* Hoffman recommends the use of them in *fevers* and *consumptions;* and Linnæus says, that by eating plentifully of them every day, he kept himself from the gout. A distilled water or wine, as well as the fruit itself, may be used in cases of the stone, and a syrup in fevers.—*Lightfoot.* The berries are grateful, cooling, subacid and juicy, and have a delightful smell; eaten either alone, or with sugar, or with milk, they are universally esteemed as a most delicious fruit; taken in large quantities they seldom disagree; they promote perspiration, impart a violent scent to the urine, and dissolve the tartar upon the teeth; used largely, they have afforded relief in the *gout* and *stone;* and Hoffman mentions their having cured *consumptions.*—Sheep and goats eat the leaves; cows are not fond of them; horses refuse them.—*Withering.*

The *Coccus Polonicus,* or Polish cochineal, is often found on the roots.

20. Potentilla. *P. anserina.*

*Ang.* Silverwood. Wild tansey. Goose grass. Goose-tansey.

*Gen.*

*Gen. Desc.* Cal. ten-cleft. Petals 5. Seeds roundish, naked, wrinkled, fixed to a small juiceless, spongy, tuburated receptacle.

*Spec. Desc.* *Stem* creeping. *Leaves* winged, serrated, silvery and white underneath; *leafits* curiously folding themselves up. *Fruitstalks* with 1 flower. *Bloss.* yellow. *Receptacle* hairy. *Sides of roads, low pastures where water has stood during winter.* Bl. June, July.

*Use.* The leaves are mildly *astringent*; dried and powdered, they have been given with success in *agues*; the usual dose is a meat spoonful of the powder every three hours between the fits.—The roots in the winter time eat like parsneps.—Swine are fond of the roots. Horses, cows, goats, and swine eat the leaves; sheep refuse them. —*Withering.*

21. Potentilla. *P. reptans. Pentaphyllum. Quinquefolium. Fragara fol. quin.*

*Ang.* Cinquefoil. Five-leaved-grass.

*Gen. Desc.* *As above.*

*Spec. Desc.* *Leaves* with finger-like divisions, leafits 5, or 3, or 7, ending in purplish points. *Stem* cylindrical, creeping, striking root. *Fruit-stalks* 1 flowered. *Flower scales,* spear egg-shaped, in pairs. *Bloss.* bright yellow, *Meadow banks, road sides.* Bl. June—Sept.

*Use.* The roots have a bitterish styptic taste, and were particularly recommended by Hippocrates for the cure of *intermittents;* with which intention they are said still to be employed by the peasantry.—*Ray.*

Its medicinal quality is confined to the external or cortical part of the root; and depending merely on its *astringent* effects, it has been prescribed *chiefly* internally in *diarrhœas* and other fluxes, and externally in gargles and astringent lotions; but being much inferior in efficacy to many other plants, it is now rarely used; in large doses, however, it may be found no bad substitute for some of the other astringents.—*Woodville.* The red cortical part of the root is mildly astringent, and *antiseptic*; a decoction of it is a good gargle for loose teeth and spongy gums.—Horses, cows, goats, and sheep eat it.—*Withering.*

Tormentilla. *T. officinalis. T. erecta. Potentilla tormentilla.*

*Ang.* Tormentel. Septfoil.

*Gen. Desc.* Cal. eight-cleft. Petals 4. Seeds roundish, naked, fixed to a small juiceless receptacle.

*Spec. Desc.* *Stem* trailing, somewhat ascending. *Leaves* sitting sitting, beautiful green, 3 together. *Leafits* serrated. *Root-leaves* on leaf-stalks. Cal. 4 smaller segm. outside the other 4. *Petals* sometimes 5, fine yellow, an orange blotch at the base; claws very short. *Stam.* 14 to 18. *Pist.* 4 to 16. *Receptacle* woolly. *Moors, barren pastures.* Bl. June, Sept.

*Use.* The root, which is the only part used medicinally, has a strong styptic taste; it ranks with the most powerful vegetable astringents, and as such has long been held in great estimation by physicians; as the resin contained in it is very inconsiderable, it seems more particularly adapted to those cases, where the heating and stimulating medicines of this class are less proper, as *phthisical diarrhœas, diarrhœa cruenta,* &c. Dr. Cullen thinks " it has been justly commended for every virtue that is competent to astringents, he himself having witnessed several instances of its virtues, and particularly having found it, both by itself, and as joined with gentian, to cure *intermittents;* but it must be given in substance and largely," *M. M. ii.* 36. Rutty recommends a decoction either in wine or water, as an application to old putrid ulcers; as a restorative to the appetite, and of the tone of the stomach, and as a powerful medicine in bloody fluxes, fluor albus, and involuntary discharges of urine; it is used by some, he says, being held in the mouth, as a preservative against contagion in the epidemic dysentery, *Rutty, M. M.* 521. The root may be given in powder from half a drachm to a drachm for a dose, or more; but it is generally given in decoction, in the following form: 1 ½ oz. of the powdered root boiled in 3 pints of water to 1 quart, to which is added, towards the end of the boiling, 1 dr. of cinnamon; of the strained liquor, sweetened with 10 oz. of any agreeable syrup, 2 oz. may be taken four or five times a day. The extracts obtained by inspissation are intensely *styptic,* the spirit is more so. *Lewis, M. M.* 654. Tormentil is ordered in the *pulvis e creta compositus* of the London College. The astringency of the root is so great, that it has been substituted for oak bark in the tanning of skins for leather; and it has been observed, that the leather has been perfected in less time than when oak bark was used. *See Mus. Rust. v. ii. n.* 12. *p.* 51.—*Woodville.* The root consists of thick black tubercles, an inch or more in diameter, replete with a red juice of an astringent quality. In the Orknies they are used for tanning leather, and found superior to oak-bark; thus, they are first boiled in water, and the leather steeped in the liquor when cold. In the same islands a decoction of these roots in milk is often used with success in *diarrhœas* and *dysenteries.* Note, perhaps it might be better not to give it in dysenteries till the morbid matter be first evacuated.—A spirituous extract of the plant is recommended in the *sea scurvy* to strengthen the gums, and fasten the teeth.—*Lightfoot.* The roots are found, by farmers, very efficacious in the *dysenteries of cattle.*—They dye *red.* Cows, goats, sheep, and swine eat it; horses refuse it.—*Withering.*

23. Geum. *G. Urbanum. Caryophyllata vulgaris.*

*Ang.* Common avens, or herb-bennet.

*Gen. Desc.* Cal. ten-cleft. Petals 5. Styles terminating. Seed with a jointed awn. Rec. pillar-like.

*Spec. Desc.* *Stem* angular. *Flowers* upright, yellow. *Fruit* globular, woolly. *Awns* hooked, bare. *Root-leaves* lyre shaped. *Steml.* in threes, jagged, toothed, unequal. *Germen* hairy. *Styles* smooth, purple, a double flexure towards the end. *Woods, hedges.* Bl. July, Aug.

*Use.* The root, which is the part medicinally employed, has an aromatic and somewhat astringent taste, with a pleasant smell. Though little used in Britain, it is held in great estimation on the continent for various virtues, but chiefly as a *febrifuge*; numerous testimonies are borne by foreign physicians of its great efficacy in obstinate *intermittents,* many of which yielded to this root, after the Peruvian bark had failed. A tincture of this root, made in the proportion of 4 oz. of it digested with a quart of brandy in a sand heat, and given in the quantity of half an ounce or more, two, three, or four times a day, is said to have very rarely failed in the cure of agues. It has been given with equal success in decoction, powder, or electuary, in the proportion in which the Cinchona bark is commonly employed. This root has also been found a useful medicine in several chronic disorders, as a general *tonic* and *astringent*; and its *antiseptic* power is proved by Buchhave. *Abs. circa. rad. Gei. urb.* to exceed that of Peruvian bark.—*Woodville.* Infused in wine it is a good *stomachic.*—The roots, gathered in spring before the stem grows up, and put into ale, give it a pleasant flavour, and prevent its going sour.—Its taste is mildly austere and aromatic, especially when it grows in warm dry situations, but in shady and moist places it has little virtue. Cows, sheep, goats, and swine eat; horses are not fond of it.—*Withering.*

( To be continued. )

